# Effect of Er:YAG laser irrigation with different etching modes on the push-out bond strength of fiber posts to the root dentine

**DOI:** 10.1007/s10103-022-03542-y

**Published:** 2022-03-14

**Authors:** Qingqing Wang, Yingmei Li, Qingfei Meng, Jian Meng, May Lei Mei

**Affiliations:** 1 College of Stomatology, Bengbu Medical College, Bengbu, China; 2 Department of Stomatology, Xuzhou Central Hospital, Xuzhou, China; 3 Department of Stomatology, Xuzhou Clinical School of Xuzhou Medical University, Xuzhou, China; 4 Sir John Walsh Research Institute, University of Otago, Dunedin, New Zealand

**Keywords:** Irrigation method, Bonding mode, Dentine, Fiber post, Push-out bond strength

## Abstract

The objective of this study was to evaluate the effect of Er:YAG laser irrigation on the push-out bond strength of fiber posts to the root dentine. Sixty extracted human mandibular first premolars were collected and decoronated. The residual roots received endodontic treatment. The treated roots were randomly divided into three groups according to different irrigation protocols: group LAI (Er:YAG laser–activated irrigation), group PUI (passive ultrasonic irrigation, positive control), and group CSI (conventional syringe irrigation, negative control) (*n* = 20). Each group was divided into two subgroups, either total-etching modes or self-etching modes (*n* = 10). After fiber post restoration, all roots were sectioned into seven 1.0-mm-thick slices. The slices received a push-out test by a universal test machine. The resin tag on the segments’ bonding interfaces was observed by scanning electron microscope. There were significant differences in the effects of the irrigation method, bonding modes, and root regions on the push-out bond strength among the groups (*p* < 0.05). The specimens with Er:YAG laser–activated irrigation and self-etching mode showed significantly the highest bonding strength (*p* < 0.001). The lengths and densities of resin tags in group PUI or group LAI with self-etching modes were longer than those in group CSI with total-etching modes. The laser-activated irrigation with self-etching modes improved the bond strength of fiber post to root dentine compared to the passive ultrasonic irrigation or conventional syringe irrigation with total or self-etching modes.

## Introduction

The fiber post has been widely used in restoring residual roots and crowns due to its promising aesthetics, biocompatibility, and mechanical properties. However, fiber posts still fail, most commonly because they debond from root dentine [1, 2]. The smear layer and dentine debris that block the dentinal tubules after a root canal treatment and the post-space preparation procedures are the main causes of debonding [3].

Effectively irrigating a root canal helps remove the smear layer, opens the obstructed dentinal tubules, and therefore increases the bond strength between the root dentine and the cement [4, 5]. The combination of the irrigation protocols of 5.25% sodium hypochlorite (NaClO) and 17% ethylene diamine tetraacetic acid (EDTA) is an effective solution for root canal irrigation because of its capability to dissolve the smear layer’s organic and inorganic content [6]. However, the smear layer cannot be completely removed with irrigates alone. Hence, the selection of irrigation methods is particularly important for removing microorganisms, the smear layer, and dentine debris from the root canal.

Laser therapy was first applied in dentistry in the 1960s and has been widely used in clinical practice, such as in treating dentine hypersensitivity, caries removal, and bleaching [7–9]. The erbium:yttrium–aluminum–garnet (Er:YAG) laser has attracted attention in dentistry in recent years. Its emission wavelength (2.940 nm) is strongly absorbed by water; it is thus effective and efficient in dental hard tissue ablation [10]. Er:YAG-activated irrigation possibly could form steam bubbles in the root canal at sub-ablative level, through which the cavitation effect is generated and the root canal is thus cleaned [11]. Previous studies have suggested that irregular dentine surfaces and dentine tubules were opened after Er:YAG laser irradiation, which increases the bonding area between the dentine and adhesive [12]. Some researchers have also confirmed that the Er:YAG laser can improve the shear bond strength between the dentine and ceramic surface and reduce microleakage [13]. However, there are few studies on the effect of Er:YAG laser on the bond strength of the fiber post to the dentine.

Conventional syringe irrigation was the most commonly used in clinical. However, there was limited efficacy because of difficulty in controlling the flow rate [14]. Passive ultrasonic irrigation transfers energy to the irrigation solution via an ultrasonically working file placed in the root canal, which can generate the “acoustic streaming effect” and “cavitation effect” in the root canal [15]. This type of irrigation can be an effective method [16]. Therefore, conventional syringe irrigation was used as the negative control while passive ultrasonic irrigation was used as the positive control in current study.

Clinicians have gradually favored universal adhesive systems because of their simplicity, which can be used in total-etching, self-etching, or selective-etching modes [17]. However, there are limited studies to compare the different bonding modes’ effects on bond strength between root dentine and the fiber post. Therefore, this study aimed to evaluate the effect of Er:YAG laser–activated irrigation with different bonding modes on the push-out bond strength of fiber posts to dentine. The null hypotheses were that Er:YAG laser–activated irrigation with different bonding modes has no improvement on push-out bond strength of the fiber post to the root dentine.

## Materials and methods

### Specimen preparation

Sixty sound human mandibular first premolars extracted for orthodontic reasons from patients aged 18–30 years who lived in the same locality were collected. Written informed consent was obtained under a protocol approved by the Ethics Committee of Xuzhou Central Hospital. After removing any attached soft tissues and examining for any dental crack stereoscopically (SMZ1500, Nikon, Japan) at 10 × magnification, the teeth were stored 0.9% saline solution at 4 °C for less than 2 weeks. The teeth were sectioned 1.0 mm coronal to the buccal cemento-enamel junction (CEJ) with an average working length of 14.0 mm. The root canals were instrumented with ProTaper instruments (K-files, Dentsply-Maillefer, Ballaigues, Switzerland) and size 4 Gates-Glidden drills (Dentsply-Maillefer) to standardize canal forms, rinsed with 2.5% sodium hypochlorite solution, dried with paper points, and then applied with a thin layer of sealer (AH Plus, Dentsply Detrey, Konstanz, Germany). The cold laterally condensed gutta percha points (Dentsply International Inc., York, PA, USA) were placed to obturate the canals, and the root canals’ coronal orifices were sealed with wax. After the endodontic treatment, all specimens were stored in 0.9% saline solution for 1 week. Subsequently, each root canal was prepared to a depth of 10 mm for no. 3 glass fiber post (#3, 3 M, USA), using matching drills with a slow-speed contra angle handpiece, according to the manufacturer’s instructions.

**Fig. 1:**
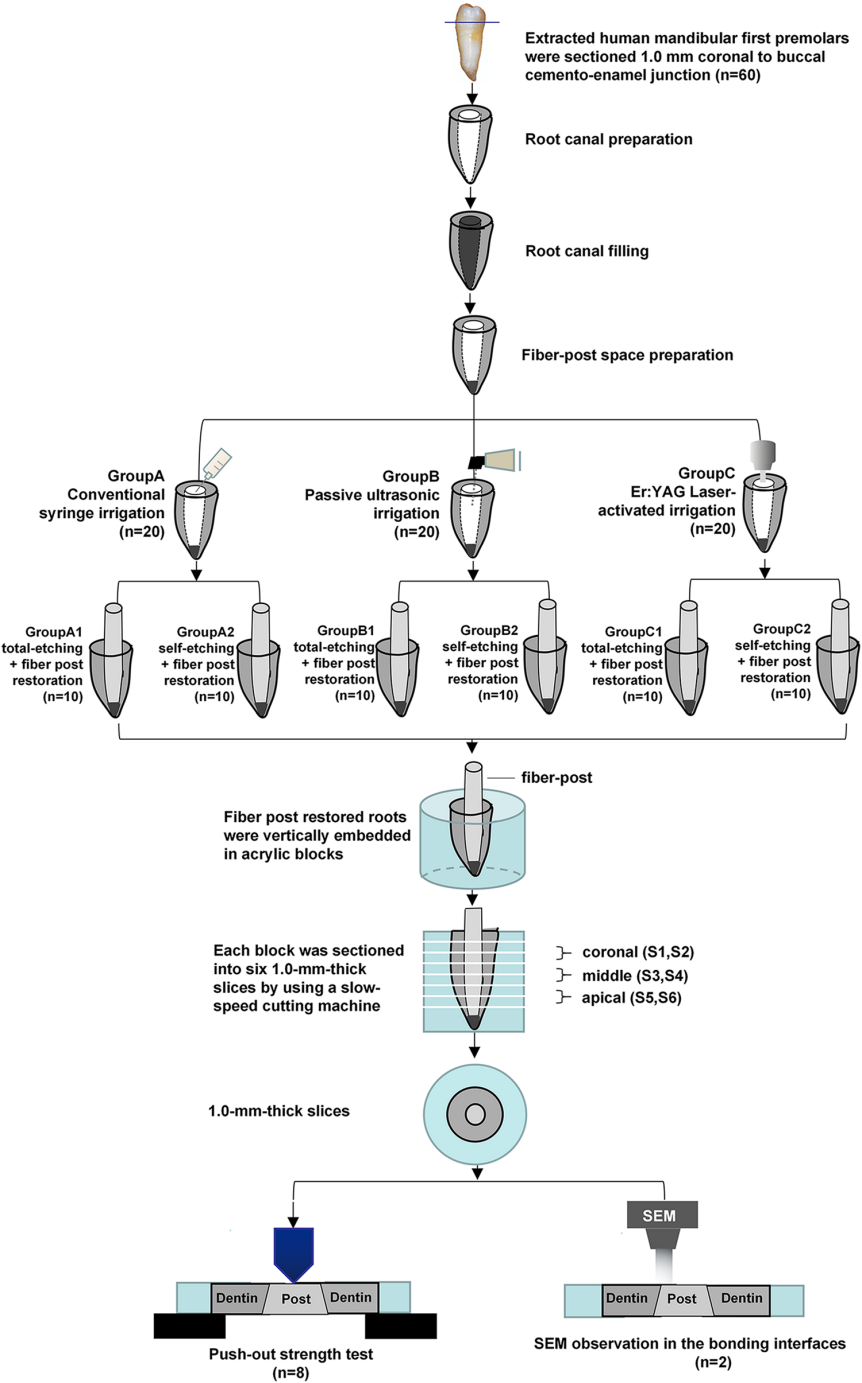
Schematic representation of the experimental process

The 60 specimens were then divided into three groups randomly (*n* = 20), according to the irrigation methods. The flowchart of the study is shown in Fig. 1.

#### Group A (conventional syringe irrigation, CSI)

The root canal was irrigated with 2 ml of 5.25% NaOCl solution and 2 ml of 17% EDTA solution for 20 s, respectively, by a conventional syringe. Subsequently, the root canal was rinsed by distilled water for 20 s. The procedures were repeated three times.

#### Group B (passive ultrasonic irrigation, PUI)

The root canal was filled by 5.25% NaOCl and 17% EDTA solution, respectively. During the passive ultrasonic irrigation process, a no. 15 ultrasonic working tip (#15, Satelec, France) of the ultrasonic treatment instrument (Satelec, France) was positioned 2 mm from the apical point of the post space and kept parallel to the root dentine wall. The ultrasound’s power was set to medium, which the manufacturer recommended for canal treatment. Each solution was activated for 20 s and then rinsed by distilled water for 20 s. The procedures were repeated three times.

#### Group C (Er:YAG laser–activated irrigation, LAI).

The Er:YAG laser (Light Walker, Fotona, Germany) (wavelength 2940 nm) was used to activate the irrigation solutions, which were the same as those in group B. During the irrigation, the 300-μm radial firing tip was injected into the post space and activated. The parameters of the Er:YAG laser were as follows: energy density 2.06 J/cm^2^, pulse energy 20 mJ, and pulse repetition rate 15 Hz. The procedures were repeated three times.

After irrigation on the post space, the specimens’ root canals were dried using absorbent paper points. Each group was randomly divided into two subgroups, according to the adhesive system’s bonding modes (total-etching or self-etching). In the total-etching mode adhesive system, the prepared root wall was first etched with 32% phosphoric acid gel (UNI-ETCH, BISCO, Inc, Schaumburg, IL, USA) for 15 s and then rinsed thoroughly with an air–water spray and dried lightly with paper points. A resin-based adhesive (Single Bond Universal Adhesive, 3 M, USA) was applied twice as a thin layer over the walls of the root wall and once over the surfaces of a prefabricated glass fiber post (#3, 3 M, USA). After thinning lightly with dry, oil-free air, the adhesive was light-cured for 10 s at 600 mW/cm^2^ (Variable Intensity Polymerizer Junior, BISCO, Inc.) [18]. The post space was filled completely by injecting resin luting cement (RelyX Ultimate Resin Cement, 3 M, USA), into which the fiber post was inserted. This was followed by light-curing for 40 s from a coronal direction. For the specimens in the self-etching mode subgroups, the fiber post cementation procedures were the same as those in the subgroups with total-etching modes except no etching process occurred in the root walls.

After 1 week in the isotonic saline storage medium, the residual roots were vertically embedded in a cylindrical block of self-cured acrylic resin (Shanghai Dental Materials Manufacture Co., Shanghai, PR China), with the fiber post parallel to the cylindrical block’s wall, and sectioned transversely with the water-cooled precision diamond saw (Isomet 1000, Buehler, USA) to obtain 1-mm-thick slices. Seven slices were obtained from each root (The length of the fiber post in the root canal is around 10 mm). The first slice from the coronal side was discarded in all groups, and the rest six slices were named from S1 to S6, respectively. According to the location of the slices in the root, S1 and S2 were considered in the coronal region, S3 and S4 in the middle region, and S5 and S6 in the apical region of the root (Fig. 1). The same operator performed all the above operations.

### Push-out bond test and failure mode

The push-out bond strengths between the fiber post and the dentine wall in the coronal, middle, and apical regions of the roots from the eight specimens in each subgroup were tested on the universal testing machine (MTS 810, USA) 2 weeks after fiber post cementation. The push-out pin was loaded at the apical part of the root slice at 0.5 mm/min crosshead speed. The maximum load at failure was recorded in Newtons (N). The bond strength was calculated through the following formula: *σ* = *C*/*A* (MPa), where *C* represents the loading at the specimen failure time (N), and *A* corresponds to the area of bonding interface (mm^2^). The bonding interface area was calculated using the following formula: *A* = *π*(*R* + *r*)[*h*^2^ + (*R* − *r*)^2^]^0.5^ (*π* = 3.14, *R* = coronal radius of the fiber post, *r* = apical radius of the fiber post, and *h* = specimen thickness) [19].

Subsequently, modes of failure were determined under a stereomicroscope at 20 × magnification. The failure modes were classified into five categories: type I, cohesive failure in dentine; type II, cohesive failure in the cement; type III, adhesive failure between the cement and the dentine; type IV, adhesive failure between the cement and the post; and type V, mixed failures consisting of a combination of two or more failure modes [20].

### Scanning electron microscopy observation of the bonding interfaces

The bonding interfaces between the cement and the dentine wall in the coronal, middle, and apical regions of the roots from the remaining 2 specimens in each subgroup were observed by SEM (Quanta™ 250 FEI, USA). The slices were treated with 32% phosphoric acid for 15 s and 5% NaOCl solution for 2 m to remove the smear layer and then dehydrated in a series of ethanol solutions (40%, 50%, 60%, 75%, 95%) for 20 m each. After coated with a gold–palladium alloy, the root slice was analyzed using SEM. The penetration lengths and the numbers of the resin tags in an SEM image selected from S1, S3, and S5 slices in each subgroup were measured and analyzed.

### Statistical analysis

The data of the push-out bond strength measured in each subgroup and in each of the root’s regions were statistically analyzed using split-plot ANOVA and Tukey HSD test by SPSS 24.0 software (SPSS Inc., Chicago, IL, USA). The resin tags penetrations were analyzed using the one-way ANOVA for multiple group comparisons followed by the Scheffe test for group wise comparison. The failure specimens’ mode results were analyzed by chi-square test. The significance level was set at 5%.

## Results

### Push-out bond strength and failure mode analysis

**Fig. 2:**
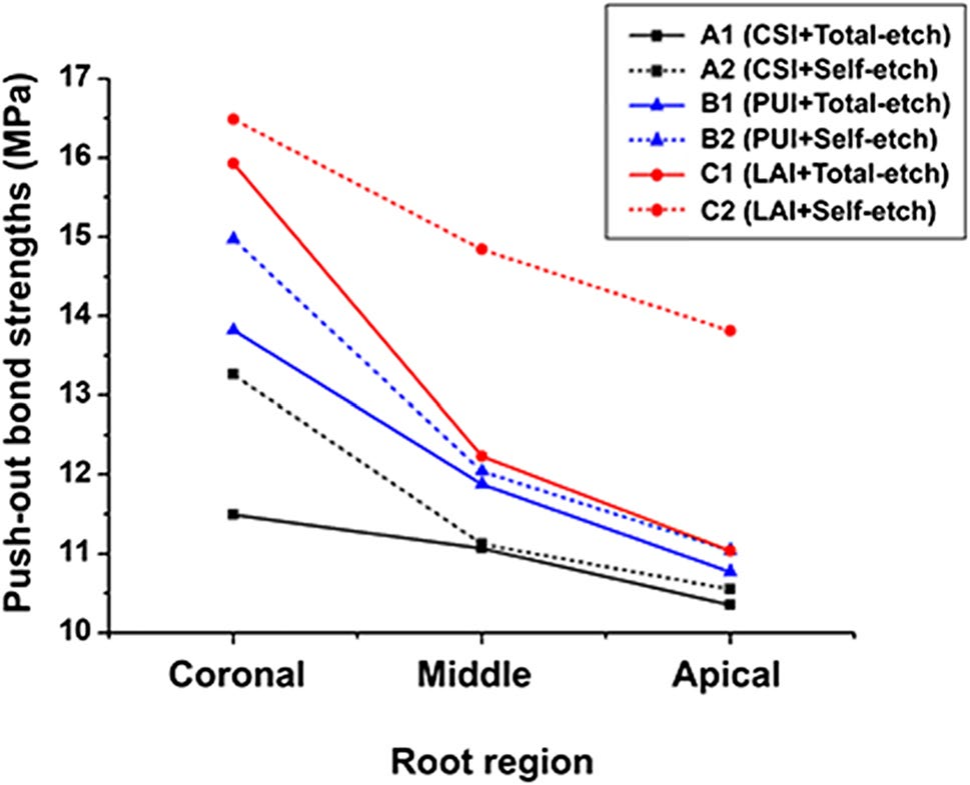
Trend chart of the push-out bond strengths of fiber posts in each group

The bond strength values for all groups are shown in Table 1 and Fig. 2. There were significant differences in the effects of irrigation methods (*p* < 0.001) and bonding modes (*p* = 0.001) on the fiber posts’ push-out bond strengths. The highest bond strength was recorded in the subgroup with laser treatment and self-etch bonding mode, and the lowest was shown in the subgroup with conventional syringe irrigation and total-etch bonding mode. Furthermore, the fiber post’s push-out bond strength decreased gradually from coronal to apical regions of the root in all groups (*p* < 0.001).

**Table 1:** Mean push-out bond strengths of fiber posts in each group (mean ± MPa)

Irrigation type	Bonding mode	Root			Total	*p* value	Multiple comparison
		Coronal	Middle	Apical			
A (CSI)	1 (Total-etch)	11.49 ± 0.40 (A1C)	11.07 ± 0.39 (A1M)	10.35 ± 0.60 (A1A)	10.97 ± 0.66 (A1)	< 0.001	A1C, A1M > A1A
	**2** **(Self-etch)**	13.26 ± 0.85 (A2C)	11.12 ± 0.33 (A2M)	10.55 ± 0.89 (A2A)	11.64 ± 1.3 (A2)	< 0.001	A2C > A2M > A2A
**B** **(PUI)**	**1** **(Total-etch)**	13.82 ± 0.49 (B1C)	11.88 ± 0.88 (B1M)	10.77 ± 0.84 (B1A)	12.16 ± 1.48 (B1)	< 0.001	B1C > B1M > B1A
	**2** **(Self-etch)**	14.97 ± 0.79 (B2C)	12.04 ± 0.61 (B2M)	11.04 ± 0.60 (B2A)	12.68 ± 1.82 (B2)	< 0.001	B2C > B2M > B2A
**C** **(LAI)**	**1** **(Total-etch)**	15.93 ± 0.40 (C1C)	12.23 ± 0.80 (C1M)	11.04 ± 0.51 (C1A)	13.06 ± 2.20 (C1)	< 0.001	C1C > C1M > C1A
	**2** **(Self-etch)**	16.49 ± 0.61 (C2C)	14.84 ± 0.51 (C2M)	13.81 ± 0.33 (C2A)	15.05 ± 1.23 (C2)	< 0.001	C2C > C2M > C2A
**Total**		14.32 ± 1.80 (C)	12.19 ± 1.40 (M)	11.26 ± 1.33 (A)			C > M > A
***p* value**		< 0.001	< 0.001	< 0.001	< 0.001		
**Multiple comparison**		A1C < A2C, B1C < B2C, C1C, C2C; A1C < B1C, A1C < C1C, B1C < C1C; A2C < B2C, A2C < C2C, B2C < C2C	A1M, A2M, B1M, B2M, C1M < C2M; A1M, B1M, A1M < C1M, B1M, CIM; A2M < B2M, A2M < C2M, B2M < C2M	A1A, A2A, B1A, B2A, C1A < C2A; A1A, B1A, A1A, C1A, B1A, C1A; A2A, B2A, A2A < C2A, B2A < C2A	A1, A2, B1, B2, C1 < C2; A1 < B1, A1 < C1, B1 < C1; A2 < B2, A2 < C2, B2 < C2		

^*^Results of Tukey HSD test comparisons were shown as superscripts, and values having same letters were not significantly different (*p* > 0.05)

Failure modes are presented in Table 2. The mode of mixed failure mainly occurred in groups B and C, while the mode of adhesive failure between the cement and the dentine mainly occurred in group A. Furthermore, the proportion of failure mode at the dentine‒cement interface decreased gradually from subgroups A1 to C2, with the proportion of the mode of mixed failure increased in the meantime. The results of chi-square test showed that the differences in the failure modes of the specimens in each group were statistically significant (*p* = 0.03).

**Table 2:** The mean mode of failures with percentages in each group

Mode of failure*	A(CSI)		B(PUI)		C(LAI)		Total
	1 (Total-etch)	2 (Self-etch)	1 (Total-etch)	2 (Self-etch)	1 (Total-etch)	2 (Self-etch)	
**I**	2 (4.2%)	2 (4.2%)	4 (8.3%)	2 (4.2%)	6 (12.5%)	6 (12.5%)	22 (7.6%)
**II**	4 (8.3%)	4 (8.3%)	-	2 (4.2%)	-	-	10 (3.5%)
**III**	24 (50%)	22 (45.8%)	18 (37.5%)	14 (29.2%)	12 (25%)	10 (20.8%)	100 (34.7%)
**IV**	8 (16.7%)	8 (16.7%)	10 (20.8%)	10 (20.8%)	12 (25%)	12 (25%)	60 (20.8%)
**V**	10 (20.8%)	12 (25%)	16 (33.3%)	20 (41.7%)	18 (37.5%)	20 (41.7%)	96 (33.3%)
**Total**	48	48	48	48	48	48	288

^*^Mode of failure: type I, cohesive failure in dentine; type II, cohesive failure in the cement; type III, adhesive failure between the cement and the dentine; type IV, adhesive failure between the cement and the post; type V, mixed failures consisting of a combination of two or more failure modes

### Resin tag measurement

**Fig. 3:**
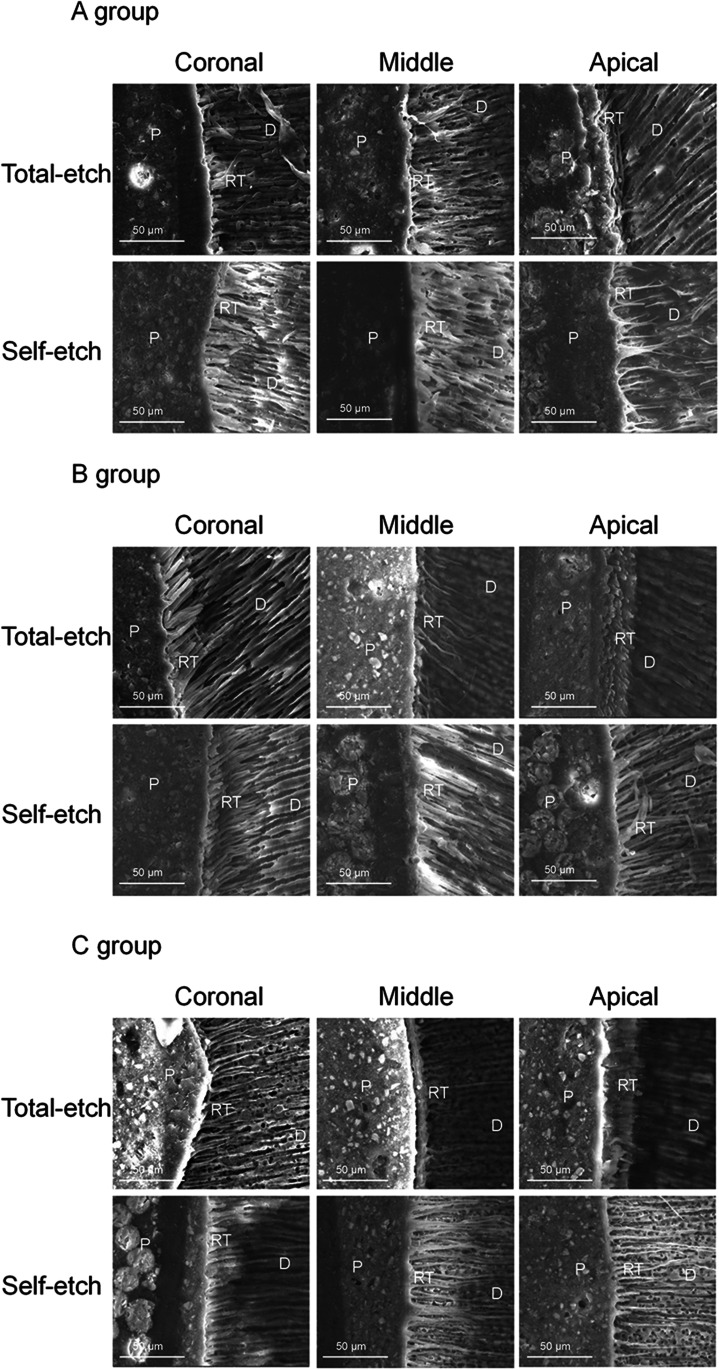
SEM images of the bonding interface in the coronal, middle, and apical regions of the roots in groups A, B and C (× 2000) (*P*, fiber post; *RT*, resin tags; *D*, root dentine)

The SEM images showed that the lengths and densities of the resin tags in groups B and C and in the subgroups with self-etching modes (groups A2, B2 and C2) were more than that in group A and in the subgroups with total-etching modes (groups A1, B1, and C1), respectively. In addition, the resin tags’ diameters in group B were more uniform and orderly arranged, while the resin tags in group C were a little thicker than those in group B. They also were melted and connected with each other in the dentinal tubules’ orifices, which gradually became thinner in the deep layer. The resin tags’ lengths and densities also decreased gradually from the coronal to apical regions of the roots in each group (Fig. 3). There were significant differences in the length of resin tag penetration (*p* < 0.001; Table 3). The density of resin tags in groups B and C were more than that in group A, and the self-etching subgroup was more than that of the total-etching subgroup, but there was no significant difference between B1 and C1.

**Table 3:** Comparison of resin tag penetration in different groups

Group	Resin tag number	Resin tag length Mean ± SD (μm)	*p* value*	Significant pairs**
A1	32	11.38 ± 6.23	*p* < 0.001	A1 and B1, A1 and B2, A1 and C1, A1 and C2, A2 and C1, A2 and C2, B1 and C2, B2 and C2, C1 and C2
A2	52	19.66 ± 6.71		
B1	59	24.85 ± 12.80		
B2	71	26.24 ± 10.49		
C1	58	29.20 ± 11.54		
C2	97	50.82 ± 21.78		

^*^One-way ANOVA comparison on resin tag length

^**^Scheffe test

## Discussion

According to this study’s results, the null hypotheses that the fiber posts’ push-out bond strength to the root dentine would not be affected by the irrigation methods or by the bonding modes of the universal adhesive system were rejected.

Effectively cleaning the dentine surface of the root canal cavity helps to improve the bond strength between the fiber post and the dentine. During the process of preparing the post space, the debris and smear layer were produced and remained on the dentine surface or within the dentinal tubules, which should be irrigated and rinsed thoroughly. Compared with the passive ultrasonic irrigation and Er:YAG laser–activated irrigation methods, the conventional syringe irrigation method’s effectiveness was less effective in irrigation, due to limited contact area between the tip of the syringe and the dentine surface [21, 22]. Recently, several types of lasers were used for irrigating root canals, such as the Er:YAG laser, the Nd:YAG laser, and the carbon dioxide laser. The wavelength of the Er:YAG laser is 2940 nm, which water and hydroxyapatite highly absorb. When it is applied on the dentine surface, the water and the hydroxyapatite of the dentine in the root canal could absorb the Er:YAG laser’s energy, causing dentine’s expansion and microexplosion, and the debris and smear layer in the root canal could be removed better than with other lasers [11, 23, 24]. In this study, the SEM observation showed that the smear layer in the dentine surface was removed more effectively by the irrigation method of passive ultrasonic irrigation or Er:YAG laser–activated irrigation combined with the irrigation protocols of 5.25% NaClO and 17% EDTA solutions than that by conventional syringe irrigation method. Furthermore, the lengths and the resin tags’ densities and coarseness in the dentine tubules in the groups irrigated by the passive ultrasonic irrigation or Er:YAG laser–activated irrigation method were found better than by the conventional syringe irrigation method. Additionally, the fiber posts’ push-out bond strength in the passive ultrasonic irrigation or Er:YAG laser–activated irrigation group was also significantly higher than that in the conventional syringe irrigation group, which may be due to the effective micromechanical binding force, chemical binding force, and friction force formed between the resin tag and the dentine tubule. In addition, as can be seen from the mean mode of failures with percentages in each group (Table 2), the damage ratio of the dentine–resin cement interface gradually decreased from the conventional syringe irrigation group to the Er:YAG laser–activated irrigation group, which fully demonstrated the significant advantages of passive ultrasonic irrigation or Er:YAG laser–activated irrigation in cleaning root canals and improving the fiber posts’ bond strength.

Using passive ultrasonic irrigation and Er:YAG laser–activated irrigation for root canal irrigation has become increasingly widespread in recent years due to their effect on irrigants. However, the differences between the two methods in removing the smear layer and improving the fiber posts’ bond strength are still controversial. Akyuz et al. [23] indicated that Er:YAG laser–activated irrigation improved the bond strength of dentine better than passive ultrasonic irrigation. However, Keles et al. [25] demonstrated that there was no significant difference in removing the smear layer and cleaning the dentine surface. According to our study’s results, the reason why the resin tags produced by passive ultrasonic irrigation were more symmetrical and orderly is because they produce vortices in the root canal and do not damage the dentine, while the Er:YAG laser has ablation and microblasting effects on the dentine surface. Furthermore, the dentine surface resin tags are thicker, some are in a molten state, and the mechanical binding force produced by them may be better than that of the passive ultrasonic irrigation treatment group. The results may explain why the fiber posts’ push-out bond strength in the Er:YAG laser–activated irrigation group is higher than that in passive ultrasonic irrigation group.

Dentine adhesive has two common formats, namely total-etching and self-etching techniques. Total-etching adhesive removes the smear layer from the dentine surface by the processes of phosphoric acid etching, allowing the adhesive to penetrate into the dentine tubules to form adhesions. However, self-etching adhesive dissolves the smear layer of the dentine without removing it, achieving coupling and demineralization while also producing a hybrid layer and resin tags [26]. Initially, total-etching adhesive technology, a three-step process (etch-rinse-adhesives), has long been considered the gold standard for adhesive systems. It remains controversial whether adding an acid-etching pretreatment while using the universal adhesives (8th generation) will improve bond strength. Chen et al. [27] indicated that the bond strength of the recent universal adhesive could not be improved by adding acid etching pretreatment. However, Rosa et al. [28] and Shafiei et al. [20] concluded that the acid etching pretreatment improved the bond strength of ultra-mild universal adhesives (such as All-Bond Universal) to dentine and was ineffective for other mild universal bonding agents. Conversely, other researchers had reported higher shear bond strength for Single Bond Universal Adhesive in a self-etching mode than a total-etching mode [29], which were generally in agreement with our results.

Generally, the dentine wall of the root canal has already obtained a clean surface after irrigation. Thus, the subsequent deepening of resin adhesive and resin tag formation will be effectively guaranteed. If acid etching pretreatment was added, etchants are difficult to be rinsed thoroughly in a limited root canal space, and keeping the moisture on the dentine surface can also be challenging. These factors could adversely affect the bond strength of total-etching adhesive to dentine. In addition, SEM images in this study illustrated that resin tags formed by self-etching mode were more noticeable than those formed by the total etching mode in each irrigation group. Hence, a higher push-out bond strength of the fiber post and a lower fracture ratio of the dentine-cement interface were shown in groups with self-etching modes than those in total-etching modes, as shown in Tables 1 and 2.

Additionally, chemical binding force is also part of the bond strength between resin and dentine. 10-Methacryloyloxydecyl dihydrogen phosphate (10-MDP) is the functional monomer in Single Bond Universal adhesives. A hydrophilic phosphate group is located at one end of 10-MDP, which combines with dentine hydroxyapatite to form calcium phosphate. A hydrophobic methacrylate group at the other end can be combined with resin. It was evidenced that the chemical binding forces between 10-MDP and calcium ions in dentine were responsible for forming the hydrophobic nanolayers and therefore improve the bond strength [30]. We found that the bond strength of the fiber post to the dentine declined gradually from the coronal to the apical region of the root (Table 1), which may be due to less dentine tubules, lower tubule density, and more difficult in removing residual sealants in the apical region than that in the coronal region [31, 32].

## Conclusions

Within the limitations of the present study, it was concluded that the bond strength of fiber post to the dentine could be improved by Er:YAG laser–activated irrigation compared to passive ultrasonic irrigation or conventional syringe irrigation techniques and that the adhesive of Single Bond Universal Adhesive using self-etching mode could increase the bond strength compared to total-etching mode, regardless of the root regions or irrigation methods.
